# IL15RA and SMAD3 Genetic Variants Predict Overall Survival in Metastatic Colorectal Cancer Patients Treated with FOLFIRI Therapy: A New Paradigm

**DOI:** 10.3390/cancers13071705

**Published:** 2021-04-03

**Authors:** Elena De Mattia, Jerry Polesel, Rossana Roncato, Adrien Labriet, Alessia Bignucolo, Sara Gagno, Angela Buonadonna, Mario D’Andrea, Eric Lévesque, Derek Jonker, Félix Couture, Chantal Guillemette, Erika Cecchin, Giuseppe Toffoli

**Affiliations:** 1 Experimental and Clinical Pharmacology, Centro di Riferimento Oncologico di Aviano (CRO) IRCCS, via Franco Gallini n. 2, 33081 Aviano, Italy; edemattia@cro.it (E.D.M.); rroncato@cro.it (R.R.); alessia.bignucolo@cro.it (A.B.); sgagno@cro.it (S.G.); gtoffoli@cro.it (G.T.); 2 Unit of Cancer Epidemiology, Centro di Riferimento Oncologico di Aviano (CRO) IRCCS, via Franco Gallini n. 2, 33081 Aviano, Italy; polesel@cro.it; 3 Pharmacogenomics Laboratory, Centre Hospitalier Universitaire de Québec (CHU de Québec) Research Center and Faculty of Pharmacy, Laval University, Québec, QC G1V 4G2, Canada; adrien.labriet.1@ulaval.ca (A.L.); chantal.guillemette@crchudequebec.ulaval.ca (C.G.); 4 Medical Oncology Unit, Centro di Riferimento Oncologico di Aviano (CRO) IRCCS, via Franco Gallini n. 2, 33081 Aviano, Italy; abuonadonna@cro.it; 5 Medical Oncology Unit, “San Filippo Neri Hospital”, Via Giovanni Martinotti, 20, 00135 Rome, Italy; mariorosario.dandrea@aslroma4.it; 6 CHU de Québec Research Center and Faculty of Medicine, Laval University, Québec, QC G1V 4G2, Canada; eric.levesque@crchuq.ulaval.ca (E.L.); felix.couture.1@ulaval.ca (F.C.); 7 Division of Medical Oncology, Ottawa Hospital Research Institute, University of Ottawa, 501 Smyth Road, Ottawa, ON K1H 8L6, Canada; djonker@toh.on.ca

**Keywords:** IL15RA, SMAD3, survival, FOLFIRI, colorectal cancer, immune system, genetic score, polymorphisms

## Abstract

**Simple Summary:**

There is an increasing scientific interest in the study of the interaction between the immune system and drugs in cancer that can affect the efficacy of an anti-cancer treatment. This study was undertaken to better understand if the genetic characteristic of a cancer patient’s immune system can predict the tumor response to the treatment and the duration of survival. The topic was studied on 335 metastatic colorectal cancer patients treated with a first-line chemotherapy (FOLFIRI regimen, irinotecan-5-fluorouracil-leucovorin). The research highlighted two markers, *IL15RA*-rs7910212 and *SMAD3*-rs7179840, significantly associated with the patient’s survival. When considering *IL15RA*-rs7910212 and *SMAD3*-rs7179840 in combination with other two genetic markers previously investigated (*NR1I2*-rs1054190, *VDR*-rs7299460), we built up a highly predictive genetic score of survival. The herein identified markers must be further validated, but still represent good candidates to understand how much a patient with a metastatic colorectal cancer can benefit from a chemotherapy with FOLFIRI regimen.

**Abstract:**

A new paradigm in cancer chemotherapy derives from the interaction between chemotherapeutics, including irinotecan and 5-fluorouracil (5-FU), and the immune system. The patient’s immune response can modulate chemotherapy effectiveness, and, on the other hand, chemotherapeutic agents can foster tumor cell immunogenicity. On these grounds, the analysis of the cancer patients’ immunogenetic characteristics and their effect on survival after chemotherapy represent a new frontier. This study aims to identify genetic determinants in the immuno-related pathways predictive of overall survival (OS) after FOLFIRI (irinotecan, 5-FU, leucovorin) therapy. Two independent cohorts comprising a total of 335 patients with metastatic colorectal cancer (mCRC) homogeneously treated with first-line FOLFIRI were included in the study. The prognostic effect of 192 tagging genetic polymorphisms in 34 immune-related genes was evaluated using the bead array technology. The *IL15RA* rs7910212-C allele was associated with worse OS in both discovery (HR: 1.57, *p* = 0.0327, Bootstrap *p*-value = 0.0280) and replication (HR: 1.71, *p* = 0.0411) cohorts. Conversely, *SMAD3* rs7179840-C allele was associated with better OS in both discovery (HR: 0.65, *p* = 0.0202, Bootstrap *p*-value = 0.0203) and replication (HR: 0.61, *p* = 0.0216) cohorts. A genetic prognostic score was generated integrating *IL15RA*-rs7910212 and *SMAD3*-rs7179840 markers with inflammation-related prognostic polymorphisms we previously identified in the same study population (i.e., PXR [*NR1I2*]-rs1054190, *VDR*-rs7299460). The calculated genetic score successfully discriminated patients with different survival probabilities (*p* < 0.0001 log-rank test). These findings provide new insight on the prognostic value of genetic determinants, such as *IL15RA* and *SMAD3* markers, and could offer a new decision tool to improve the clinical management of patients with mCRC receiving FOLFIRI.

## 1. Introduction

The cooperation between chemotherapeutic agents and immune system aiming at eradicating tumors represents a new paradigm in cancer chemotherapy. The cancer-related immune response impacts the efficacy of chemotherapy by multiple mechanisms, including the modulation of the chemotherapy-mediated tumor cell death [1]. On the other hand, chemotherapeutic agents can induce immunogenic cell death (ICD), making the cancer cells more immunogenic and stimulating an anti-tumor immune response mediated by effectors T-cell [2]. Among chemotherapeutics, irinotecan and 5-fluorouracil (5-FU) have a significant immune-modulatory effect, influencing the overall antitumor response and disease outcome [3,4,5,6,7]. These findings open up a novel field of immunogenetic investigation aimed at defining the role of the host variability in immune-related genes in predicting the response to treatment and patients’ prognosis.

The cancer-related inflammation response is also indicated to impact the efficacy of chemotherapy by regulating the inflammation-related transcriptional factors and in turn the expression of drug metabolic genes [8,9,10]. We have previously reported significant associations between genetic variants in inflammation-related transcriptional regulators and clinical outcome after administration of FOLFIRI (irinotecan, 5-FU, leucovorin) [11,12,13]. Particularly, pregnane X receptor (PXR; *NR1I2*) rs1054190 and vitamin D receptor (*VDR)* rs7299460 polymorphisms emerged as prognostic markers of OS for metastatic CRC (mCRC) patients receiving FOLFIRI [12].

FOLFIRI regimen represents a cornerstone of systemic treatment for mCRC [14,15,16], due to the significant survival advantage reported by clinical trials [17,18]. The more recent combination of FOLFIRI with molecularly targeted drugs [14,15,16], has further enhanced the efficacy of mCRC therapy with a survival benefit [15]. Nonetheless, despite these improvements, the 5-year survival rate continues to be low (~14%) [15], and the inter-individual heterogeneity observed in the therapy outcome still represents a crucial problem in the clinical management of patients with mCRC. Over the last years, huge pharmacogenetic research efforts were made to identify validated predictive markers of the response to FOLFIRI-based therapy in mCRC setting. Specific attention was posed on genes related to drug metabolism and mechanism of action [19,20]. However, although a number of genetic markers were significantly associated with irinotecan- or fluoropyrimidine-related toxicity and entered into clinical guidelines (i.e., UGT1A1*28; *DPYD* variants) [19,21], validated germline genetic markers that could predict FOLFIRI effectiveness and patients’ survival have yet to be identified [19,22,23]. Some biochemical (i.e., carcinoembryonic antigen, carbohydrate antigen 19-9 and 125) [24,25] and clinical (i.e., primary tumor site) [14,26] parameters as well tumor features (i.e., *RAS*, *NRAS*, and *BRAF* mutations; microsatellite instability/mismatch repair status) [14,15,16] have been reported to play a role in predicting efficacy and patients’ prognosis after the combination of FOLFIRI with targeted agents. However, despite these promising findings, a significant variability in the clinical outcome is still present. Therefore, the evaluation of the host genetic profile could contribute to better stratify patients who undergo therapy for mCRC on the basis of the treatment outcome.

The present work was designed to discover potential genetic markers of overall survival (OS) by analyzing genes encoding proteins involved in the immune system and related networks. The study, adopting a discovery/replication design and including 335 mCRC FOLFIRI-treated patients, evaluated 192 tagging polymorphisms (TagSNP) in 34 immune-related genes. The primary aim was to identify novel genetic prognostic markers that could improve the pre-treatment identification of patients who may benefit from a first-line FOLFIRI administration. The secondary endpoint was to integrate these genetic markers related to the immune system with those related to the inflammation response (i.e., *NR1I2*-rs1054190, *VDR*-rs7299460), that we previously identified in the same study population [12], to generate a genetic score of OS that could further improve the clinical management of patients with mCRC.

## 2. Patients and Methods

### 2.1. Patient Cohorts and Treatment

The study was performed retrospectively on prospective cohorts and includes a total of 335 patients with mCRC receiving first-line FOLFIRI regimen sub-grouped into a discovery and a replication cohort.

The discovery cohort, previously described [27,28], included prospectively enrolled North-Eastern Italian patients homogenously treated between February 2002 and November 2005 [28]. Information on survival and progression was obtained through an active follow-up. While all the 250 eligible patients had OS records, for 21 patients progression data were missing. All patients received 180 mg/m^2^ intravenous dose of irinotecan in FOLFIRI regimen (Tournigand-modified FOLFIRI regimen [29] in most patients). The detailed definition of the criteria of eligibility, treatment decision check-points, as well as the methods for efficacy assessment and data classification have been previously reported [28].

The replication cohort comprised 92 patients with mCRC prospectively enrolled at three medical centers in Eastern Canada from 2003 to 2012 [27]. All patients were treated with FOLFIRI regimen and received 180 mg/m^2^ intravenous dose of irinotecan every 2 weeks. More information about eligibility criteria, treatment modalities, and clinical data collection were published elsewhere [13,30]. Survival and progression data were obtained through an active follow-up. OS and progression free survival (PFS) data were available for all 92 eligible patients included in the study.

The study was conducted according to the ethical guidelines of the 1975 Declaration of Helsinki and was approved by the Comitato Etico Indipendente-Centro di Riferimento Oncologico di Aviano and the CHU de Quebec ethics committees. All patients signed a written informed consent for research purposes before entering the study.

### 2.2. Marker Selection

Candidate genes were selected based on a literature search (PubMed-MEDLINE) focusing on those encoding for cytokines, chemokines, and all the related proteins involved in the regulation of the immune response signaling network in colorectal cancer (CRC). Genes regulating the activation of the immune system against the cancer antigens generation stimulated by the chemotherapeutic agents (as irinotecan or 5-FU) were also considered. Successively, genetic variants for each candidate gene were selected using a TagSNPs approach that allowed covering the genetic diversity of targeted genes. Each TagSNP captures a block of linked polymorphisms at a stringency of r^2^ = 0.80. A detailed description of the bioinformatic workflow for the selection of TagSNPs has been previously reported [31]. At the end of the bioinformatics workflow, a cohort of 192 molecular markers in 34 candidate genes (Supplementary Table S1) were selected and introduced into the genetic analysis.

### 2.3. Genetic Analysis

#### 2.3.1. Discovery Cohort

Genomic DNA was extracted from peripheral blood samples using the automated extractor BioRobot EZ1 and the Kit “EZ1 DNA Blood Kit 350μL” (Qiagen Inc., Valencia, CA, USA). Genotyping was performed on an Illumina BeadXpress platform (Illumina, Inc., San Diego, CA, USA) based on Golden Gate chemistry. A 192-plex Illumina VeraCode GoldenGate Genotyping Assay was developed using the “Assay Design Tool” available on the Illumina website [32]. A detailed description of the bioinformatic workflow for assay design, analytical procedures, data collection and analysis, and data quality control have been previously published [31]. Sample replicates were introduced into each analysis. Only the DNA samples and polymorphisms with a call rate > 90% were retained in the final report.

#### 2.3.2. Replication Cohort

An iPLEX matrix-assisted laser desorption/ionization time-of-flight mass spectrometry method (Sequenom, San Diego, CA, USA) was employed to detect the genetic variants in patients belonging to the replication cohort. The Spectro DESIGNER software (Sequenom, San Diego, CA, USA) was used to design all extension primers and PCR assays. Negative controls and a 5% random sample duplicate population were used to ensure robustness and reproducibility of the analysis.

### 2.4. Bioinformatic Analysis

A functional prediction to determine the putative effect of the polymorphisms selected through the statistical analysis was performed using three online softwares: HaploReg v4.1 [33], RegulomeDB v2.0 [34], and Ensembl’s Variant Effect Predictor (VEP) Ensembl release 102—November 2020 [35].

HaploReg v4.1 was employed to test the functional effect of a selected polymorphism and all the others included in the same haploblock at a stringency of r^2^ = 0.80 using the linkage disequilibrium data from 1000 Genomes Project (EUR). This tool allows exploring annotations of the non-coding variants. HaploReg includes the chromatin state and protein binding annotation, sequence conservation across mammals, effect of polymorphisms on regulatory motifs, and effect of polymorphisms on expression from expression quantitative trait locus (eQTL) studies.

RegulomeDB v2.0 is a database that annotates polymorphisms in the intergenic regions of the human genome by integrating a big collection of regulatory information from several public datasets. This tool returns two scores: a rank score (1 to 7) with the lower value indicating the stronger evidence for a variant to be in a functional region; and a probability score (0 to 1), with 1 being most likely a regulatory variant. The scores are assigned by integrating annotation data on the methylation profile, chromatin structure, protein motifs, binding to transcription factors, and enhancer activity.

VEP Ensembl was used to determine the effects of the genetic variants on genes, transcripts, and protein sequences, as well as regulatory regions. The potential functional impact is described through a number of predictive scores including the Combined Annotation Dependent Depletion (CADD) score that integrates multiple annotations into one metric proportionally ranking the variants by deleteriousness [36]. The VEP tool allows annotating the sequence variants that are located not only in the non-coding region of the gene but also in the coding sequence of the protein and provides data on the associated phenotype, clinical value, and literature evidences.

### 2.5. Statistical Analysis

**Figure 1:**
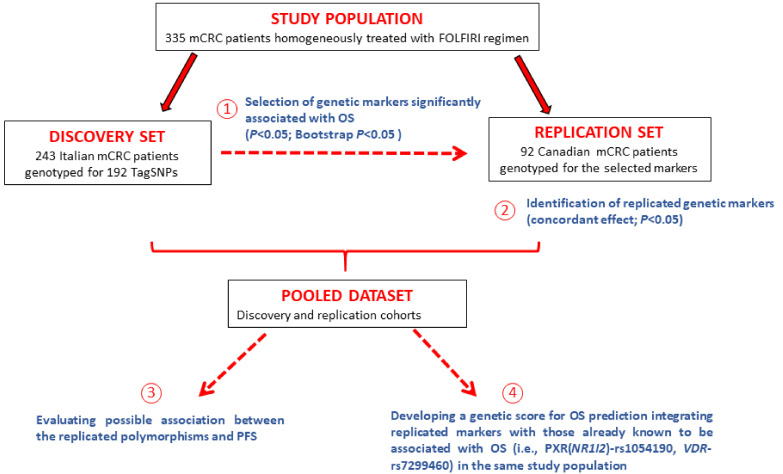
Study design.

For each patient, the time at risk was calculated from treatment initiation to death, progression (for PFS only), or last follow-up, whichever came first. The statistical analysis and study design can be summarized in four main steps (Figure 1).

(1) Selection of potential polymorphisms significantly associated with OS in the discovery cohort. The association between polymorphisms and OS was evaluated by calculating the hazard ratio (HR) of death and corresponding 95% confidence interval (CI) in a Cox proportional hazards model. HRs were adjusted for gender, age, cancer site, stage at diagnosis, radical surgery, and adjuvant chemotherapy, when available. Dominant, recessive, and additive genetic models were considered for each polymorphism by combining heterozygous with homozygous genotypes; the best-fitting genetic model was selected according to the Wald chi-squared test. A significant association (*p* < 0.05) was tested for robustness through a bootstrap procedure with 1000 re-sampling.

(2) Testing the selected polymorphisms in an independent replication cohort. Each polymorphism significantly associated to OS in the discovery cohort was further replicated in an independent replication cohort according to the following hierarchical approach: (a) The association between the polymorphism and OS was estimated according to the same genetic model found in the discovery cohort; (b) when the HRs from the discovery and replication cohorts were discordant (i.e., HRs with different directions), the polymorphism was considered as “not replicated” and the validation process stopped; (c) when HRs were concordant, the HR from the Cox proportional hazard model in the replication cohort was tested for significance using a one-tailed Wald χ^2^ test; replication was claimed for *p* < 0.05. The discovery and the replication datasets were then combined to estimate the OS probabilities, according to genetic polymorphisms, by the Kaplan-Meier method; survival differences were tested using the log-rank test. 

(3) Evaluating a possible association between the selected polymorphisms and PFS. Polymorphisms significantly associated to OS (steps 1 and 2) were further investigated in the pooled dataset to evaluate their relationship with PFS according to the Kaplan-Meier method.

(4) Developing a genetic score for OS prediction. Genetic markers identified in the previous steps were integrated with those already known to be associated with OS (i.e., *NR1I2*-rs1054190, *VDR*-rs7299460) in the same study population [12], using the pooled dataset. For each polymorphism, point scores, according to the risk of death emerging from the Cox model including all genetic variants as covariates, were assigned to the presence of risk alleles, 0 otherwise. The point scores for each polymorphism were then summed up to a genetic score. Considering the low frequency of some polymorphisms (i.e., *NR1I2*-rs1054190) splitting the dataset into discovery and replication cohorts was not feasible.

## 3. Results

### 3.1. Patients and Genotyping

The average sample call rate was 0.97 (0.90–0.98), whereas the average genotype call rate was 0.99 (0.90–1.00). Twenty-nine polymorphisms failed at the quality check and were excluded from the statistical analysis. Replicated samples included in the analysis were 100% concordant. Seven out of 250 samples in the discovery cohort were eliminated since they did not reach the fixed call rate threshold of 90% probably due to low DNA quality. 

A Sequenom assay was successfully developed for all the polymorphisms to be tested in the replication cohort. All the 92 samples in the replication cohort were successfully genotyped with an average genotype call rate of 0.99 (0.95–1.00) and a sample call rate of 0.99 (0.91–1.00). 

The two study populations (discovery and replication cohorts) were well-balanced (*p* > 0.05) for all the major demographic and clinical characteristics (Table 1). All patients had a metastatic disease (stage IV) at the time of enrollment in the present study. One hundred and twelve patients had metastasis located in the liver only, 36 in the lung only, and 16 had a single site metastasis in other organs; 79 patients had metastases located in multiple organs. All patients were self-reported Caucasian.

### 3.2. Markers of Overall Survival

The discovery and replication cohorts had a similar OS pattern (*p* > 0.05, Table 1).

In the discovery cohort, 23 genetic variants in 11 immune-related proteins (FAS, FOXO3, MIF, IFNGR2, IL15RA, SMAD-3, STAT3, STAT5A, STAT6, TGFBR2, TLR10) resulted significant predictors of OS (*p* < 0.05 and Bootstrap *p*-value < 0.05): 14 out of 23 markers were associated with shorter OS (HRs: 1.32–35.31), while the remaining nine with a longer OS (HRs: 0.51–0.72) (Table 2).

The genotype distribution of the 23 selected polymorphisms is reported in Supplementary Table S2. The minor allele frequencies were in line with the data reported for the Caucasian population in the dbSNP database [37]. Deviation from the Hardy–Weinberg equilibrium was tested by the chi-squared test, and no deviation was found (*p* > 0.05) except for the variant *SMAD3*-rs3743343 in the discovery cohort and *IL15RA*-rs3136626 in the replication cohort (Supplementary Table S2).

Two out of 23 markers, *IL15RA*-rs7910212 and *SMAD3*-rs7179840, were successfully replicated (*p* < 0.05) in the Canadian cohort applying the same genetic model. The CC or CT genotype at *IL15RA*-rs7910212 was significantly associated with an increased risk of death in the discovery (HR: 1.57, *p* = 0.0327, Bootstrap *p*-value = 0.0280) and replication (HR: 1.71, *p* = 0.0411) cohorts with respect to the TT genotype. In contrast, the CC or CT genotypes at *SMAD3*-rs7179840 were associated with a lower risk of death in the discovery (HR: 0.65, *p* = 0.0202, Bootstrap *p*-value = 0.0203) and replication (HR: 0.61, *p* = 0.0216) cohorts with respect to the TT genotype.

The potential bias, due to the type of second-line treatment received by the patients after the first disease progression, was further evaluated in the discovery cohort. Overall, 199 patients experienced a disease progression, which was treated with FOLFOX in 117 (58.8%) cases and with other chemotherapeutic regimens in 61 (30.7%) cases; 21 (10.5%) patients underwent other or no treatment. After the inclusion of the type of second-line treatment in the regression model, the hazard ratio of death did not show a significant variation: HR: 1.53 (1.01–2.32) for *IL15RA*-rs7910212 and HR: 0.64 (0.45–0.93) for *SMAD3*-rs7179840.

**Figure 2:**
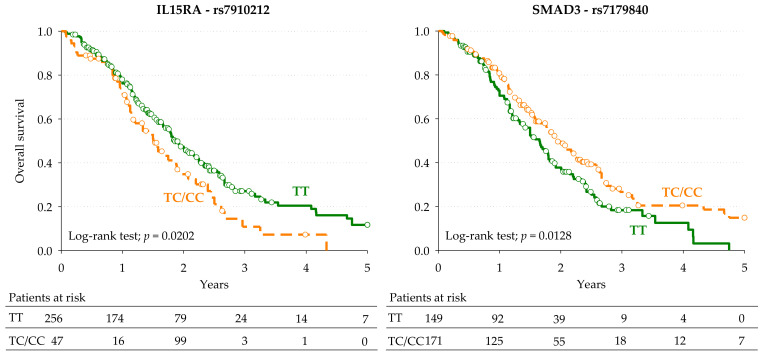
Kaplan-Meier estimates of overall survival according to the selected *IL15RA*-rs7910212 and *SMAD3*-rs7179840 polymorphisms in the combined discovery and replication cohorts (pooled population, *n* = 335).

Kaplan-Meier curves for OS according to *IL15RA*-rs7910212 and *SMAD3*-rs7179840 in the pooled population are shown in Figure 2. With regards to the *IL15RA*-rs7910212, patients carrying the minor C allele (CC or TC genotype) had a median OS of 18 months, compared to TT genotype carriers, who had a median OS of 22 months (*p* = 0.0202, log-rank test). Concerning *SMAD3*-rs7179840, patients harboring the minor C allele (CC or TC genotype) had a median OS of 23 months, compared to those with the TT genotype, who had a median OS of 19 months (*p* = 0.0128, log-rank test). These associations were confirmed in the pooled population by the multivariable Cox regression analysis (*IL15RA*-rs7910212, pooled HR: 1.55, 95% CI: 1.03–2.33, *p* = 0.0356; *SMAD3*-rs7179840, pooled HR: 0.66, 95% CI: 0.46–0.95, *p* = 0.0234).

### 3.3. Markers of Progression-Free Survival

**Figure 3:**
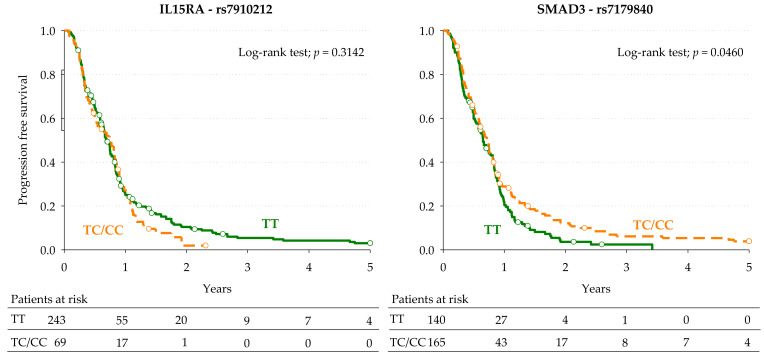
Kaplan-Meier estimates of progression-free survival according to the selected *IL15RA*-rs7910212 and *SMAD3*-rs7179840 polymorphisms in the combined discovery and replication cohorts (pooled population, *n* = 335).

Kaplan-Meier curves for PFS according to *IL15RA*-rs7910212 and *SMAD3*-rs7179840 in the pooled population are shown in Figure 3. With regards to the *IL15RA*-rs7910212 variant, the median PFS for patients carrying the minor C allele (CC or TC genotype) or the TT genotype was not statistically different (8.9 and 8.3 months, respectively; *p* = 0.3142, log-rank test). Conversely, for the *SMAD3*-rs7179840 marker, patients with the minor C allele (CC or TC genotype) had a significantly longer median PFS compared to those with the TT genotype (8.8 and 7.8 months, respectively; *p* = 0.0460, log-rank test). The impact of the *SMAD3*-rs7179840 variant on PFS was consistent with that observed on OS. The multivariable Cox regression analysis found no significant association (*IL15RA*-rs7910212, pooled HR: 1.09, 95% CI: 0.78–1.54, *p* = 0.6077; *SMAD3*-rs7179840, pooled HR: 1.11, 95% CI: 0.83–1.47, *p* = 0.4917).

### 3.4. Prognostic Score for Overall Survival

**Figure 4:**
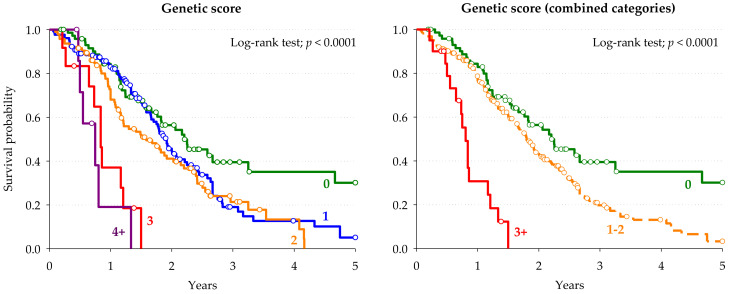
Kaplan-Meier estimates of overall survival according to the genetic score in the combined case series (*n* = 320).

A genetic prognostic score of OS combining the previously identified markers (i.e., *NR1I2*-rs1054190, *VDR*-rs7299460) [12] with those selected in the present study (i.e., *IL15RA*-rs7910212, *SMAD3*-rs7179840) was developed. A total of 320 out of 335 patients (discovery and replication cohorts) were eligible for the score development as they had the genotype data for the four candidate polymorphisms. The score point for each polymorphism was assigned based on the risk of death (i.e., HR) estimated by the Cox regression analysis (Table 3), adjusting for potential polymorphism-polymorphism interaction. A significant increase in the risk of death according to the score points was observed (Table 4). Particularly, patients carrying 1 or 2 score points had about 2-fold higher risk of death with respect to those carrying zero score points (HR: 1.90, *p* = 0.0007); even more remarkable, patients harboring more than 3 score points had about 7-fold higher risk of death with respect to those carrying zero score points (HR: 7.37, *p* < 0.0001). The capability of the genetic prognostic score to stratify patients according to the different OS outcomes was further evidenced by Kaplan-Meier curves (Figure 4). This analysis showed a strongly significant trend towards a shorter OS with increasing score points (*p* < 0.0001, log-rank test). Patients carrying “more than 3” or “1 or 2” score points had a median OS of 18.7 and 22.8 months, respectively, compared to those with zero score points, who had a median OS of 26.0 months (*p* < 0.001, log-rank test).

### 3.5. Bioinformatic Analysis of IL15RA-rs7910212 and SMAD3-rs7179840

With regards to the rs7910212 variant, located in the intron region of the *IL15RA* gene, a summary of the in silico analysis of its functional impact is reported in Supplementary Table S3A. *IL15RA*-rs7910212 was predicted to have a minimal damaging effect on the gene functionality and/or expression by the HaploReg and VEP software (i.e., CADD score = 2.490; one motif changed for regulators of gene transcription). Similarly, analyzing *IL15RA*-rs7910212 by RegulomeDB, a rank score of 5 (i.e., “minimal binding evidence” supported by transcription factors binding or DNase peak data) and a probability score of 0.13454 was achieved. The use of HaploReg showed that two additional intronic variants are tagged by *IL15RA*-rs7910212 (r^2^ > 0.8): *IL15RA*-8177685 (r^2^ = 0.97) and *IL15RA*-rs7917197 (r^2^ = 0.96). Similarly to rs7910212, both tagged polymorphisms were globally predicted to have a minor regulatory function (RegulomeDB rank score of 5) (Supplementary Table S3A). Among them, the most functionally relevant seemed to be the *IL15RA*-8177685 variant that was supposed to alter regulatory chromatin states (i.e., one enhancer histone marks items) and motifs (two motifs changed) displaying a Regulome probability score of 0.45052. *IL15RA*-8177685 was also suggested by literature data to be a functional variant impacting the immune-modulatory activity of IL15RA [38].

For the rs7179840 variant, located in the intron region of the *SMAD3* gene, the results of the bioinformatic analysis are summarized in Supplementary Table S3B. *SMAD3*-rs7179840 could have a moderate impact on the gene functionality and/or expression since it was predicted to broadly alter regulatory chromatin states (i.e., two promoter histone marks, 15 enhancer histone marks, nine DNAse items) and motifs (three motifs changed) by the HaploReg tool. This effect was summarized by a RegulomeDB rank score equal to 4 (i.e., “minimal binding evidence” supported by transcription factors binding and DNase peak data) and a probability score equal to 0.60906. The VEP tool indicated a CADD score of 0.126 and an association of rs7179840 with some pathologic phenotypes. HaploReg identified one additional non-coding polymorphism in the *SMAD3*-rs7179840 haploblock (r^2^ > 0.8), the intronic *SMAD3*-rs7183244 (r^2^ = 0.97) (Supplementary Table S3B). Similarly to rs7179840, the linked rs7183244 could have a moderate functional impact; this genetic variant was predicted to change regulatory chromatin states (i.e., one promoter histone marks, 14 enhancer histone marks, two DNAse items) and exhibit eQTL effects (i.e., one GRASP QTL hits). The RegulomeDB rank score was equal to 4 and the probability score to 0.60906. The VEP software returned a CADD score of 1.396 and a correlation with pathological phenotypes.

## 4. Discussion

It is acknowledged that the host immune system plays a crucial role in modulating the CRC development, progression, and prognosis [39,40,41,42], as well as in regulating the chemotherapy effectiveness [1,8,9,10]. Recently a new paradigm has been proposed to explain the anti-tumor effect of the chemotherapy through fostering the activation of the anti-cancer immune-system by the induction of ICD on the tumor [2]. This stimulates the exploration of genetic markers in the immuno-related pathways that could optimize the stratification of patients according to their probability to benefit from chemotherapy. The effect of 5-FU and irinotecan on the immune system has been previously reported [3,5,6,7], but its clinical impact on patients’ survival has yet to be investigated.

The main finding of the present study was the identification of *IL15RA*-rs7910212 and *SMAD3*-rs7179840 as novel genetic prognostic markers of OS after the FOLFIRI treatment, with a significant effect in two independent cohorts of patients with mCRC. 

Interleukin-15 (IL-15) represents a critical factor for the regulation of the immune response. It induces the activation of T, B, and natural killer cells, enhances the cytolitic capacity of CD8(+) T cells and avoids the stimulation of the immunosuppressive T regulatory cells [43]. IL-15α, encoded by IL15RA, is the high affinity receptor for IL-15 and is expressed on IL-15-producing cells (e.g., macrophages, monocytes, and dendritic cells) [43,44]. IL-15 is minimally secreted but shows the peculiarity to be predominantly trans-presented in conjunction with its receptor IL-15α for delivery to target cells. This unique mode of presentation confers the ability of IL-15 to mediate its distinctive functions [44,45]. Due to its impact on the immune system, the IL-15/IL-15α axis modulates the carcinogenesis process by inhibiting tumor growth [43,44,45]. Specifically, studies on in vitro and xenograft models demonstrated that IL-15 inhibits colon carcinogenesis and prolongs survival by enhancing the immune response [46,47]. Interestingly, only IL-15 trans-presented via its receptor IL-15α, but not soluble IL-15, could efficiently stimulate the cytotoxic function of immune effectors against colon carcinoma cells [48]. The IL-15/IL-15α complex was also shown to potentiate the antitumor activity of some chemotherapeutics, including 5-FU and irinotecan [49,50,51]. A study on the murine model of colon carcinoma reported the ability of IL-15 to improve the therapeutic index and antitumor efficacy of 5-FU [50]. Similar results were obtained for irinotecan, whose therapeutic efficacy against advanced CRC and liver metastases was shown to be enhanced by IL-15 in the rat models [49,51]. Although the mechanisms of interaction between IL-15 and chemotherapeutics need to be further clarified, it could be the result of the combined cytotoxic effect of drugs on tumor cells together with stimulation of an antitumor immune response by the IL-15/IL-15α complex. Thus, an altered IL-15α functionality, determined by the host genetic variation, could impact both the control of tumor growth by immunity and the FOLFIRI anti-cancer activity. This would finally affect the prognosis of patients with mCRC, as observed in the present study. *IL15RA*-rs7910212 is an intronic variant of unknown functional significance. The bioinformatic analysis was predicted for rs7910212, a damaging effect on the IL-15RA functionality and/or expression. Nonetheless, considering the limited size of this effect, it could not be excluded that the observed clinical phenotype could be related to other genetic variants located in the same haploblock (i.e., rs8177685; rs7917197), with a supposed regulatory role on the IL-15RA gene.

SMAD-3 represents a major transcription factor in transforming growth factor-β (TGF-β) downstream signaling. The TGF-β/SMAD-3 pathway plays a critical role in several biological processes including the control of cell proliferation, apoptosis, differentiation, epithelial-mesenchymal transition, and anticancer immune response [52,53]. The TGF-β/SMAD-3 signaling has been found to be implicated in CRC carcinogenesis with both tumor suppressor effects in the early development of cancer and pro-metastatic effects in late stage disease [52,53]. A high expression of TGF-β in CRC tissues has been correlated with tumor progression, neo-angiogenesis, lymph-node metastases, and immunosuppression, as well as poor prognosis and adverse clinical outcomes [53,54,55]. Genetic mutations in the TGF-β pathway genes, including *SMAD3*, contribute to the CRC aggressive phenotype [54,56]. Moreover, *SMAD3* inherited genetic variants were reported to be strongly associated with survival after diagnosis of CRC [57,58]. The TGF-β/SMAD-3 pathway has also been found to have a pivotal role in the CRC mechanisms of resistance to drugs including 5-FU [55,59,60]. An in vitro study evidenced that SMAD-3 is crucially involved in the 5-FU-resistant pathway in CRC by modulating TGF-β downstream genes with pro-proliferative, pro-metastatic, and anti-apoptotic effects [60]. Other analyses on in vivo and in vitro models of CRC reported that 5-FU is able to stimulate the activation of SMAD-3 and the related TGF-β pathway in chemoresistant cells inducing changes in the surrounding tumor microenvironment (i.e., increased vascularization) and cell mechanisms of death and proliferation [55]. On the other hand, the repression of the TGF-β signaling was shown to inhibit the transcription of 5-FU-induced genes and to restore the sensitivity of chemoresistant cells to 5-FU [55]. Therefore, similarly to IL-15, an altered SMAD-3 activity associated with specific genetic variants could modify the TGF-β-related transcriptional response impacting on both the tumor development and the antitumor FOLFIRI efficacy, finally affecting the patients’ prognosis. According to this hypothesis, in the present study, *SMAD3*-rs7179840 emerged as a prognostic marker, being associated with better OS and PFS. The functional meaning of this intronic polymorphism has not been described yet. *SMAD3*-rs7179840 as well as its linked variant rs7183244 (r^2^ = 0.97) were predicted to have a moderate impact on the gene functionality and/or expression by the in silico analysis. Thus, even if the exact functional consequences of *SMAD3*-rs7179840 variant remain to be finally elucidated, it could likely impact on SMAD3 functions. 

In the present study, the novel prognostic immune-related markers *IL15RA*-rs7910212 and *SMAD3*-rs7179840 were integrated with those previously identified in the same study population in the inflammation-related response (i.e., *NR1I2*-rs1054190, *VDR*-rs7299460) [12]. This generated a genetic risk model that significantly stratified patients with mCRC according to OS. PXR and VDR are transcriptional factors belonging to the nuclear receptor super-family. These proteins have been reported to mediate the impact of inflammation on the expression of metabolic genes and finally on therapy outcome after 5-FU and irinotecan administration [8,9,10,12]. IL15RA and SMAD-3 have been suggested to modulate the antitumor immune response and to interact with the mechanism of action of 5-FU and irinotecan. All these proteins (PXR, VDR, IL15RA, SMAD-3) have also been indicated to modulate the CRC development and aggressiveness. Thus, the risk score, combining the four independent genetic variants linked to the inflammation and immune response, could optimally integrate the impact of these markers and related pathways on both tumor biology and FOLFIRI efficacy and finally predict the patients’ prognosis. 

It should be noticed that, although FOLFIRI still represents the cornerstone of first-line treatment for patients with mCRC, it is no longer administered alone but in association with targeted agents, particularly antiangiogenics or anti-epidermal growth factor receptor (EGFR) agents. Nevertheless, the results of the present study highlighted a better chemo-responsiveness in some patients depending on their inhered genetic features, and this effect could be regardless of the combination with a targeted drug. Interestingly, recent literature data, obtained in solid tumors including colorectal carcinoma, have suggested that both IL-15/IL-15α axis and TGF-β/SMAD-3 pathway are also involved in determining the effectiveness of the anti-EGFR agent, cetuximab. Particularly, the IL-15/IL-15α axis has been reported to enhance the efficacy of cetuximab by promoting activation of both natural killer and dendritic cells [61,62,63]. Conversely, the activation of TGF-β/SMAD-3 signaling has been indicated to limit the cetuximab efficacy [64]. These preliminary results call for additional studies aiming to define the effect of *IL15RA* and *SMAD3* genetic markers in a clinical context in which FOLFIRI is used in association with anti-angiogenic or anti-EGFR agents. 

The present work has some limitations that need to be considered. First, because of the retrospective nature of the analysis, the genetic markers identified in the current study require an independent validation by further biomarkers-driven prospective clinical trials prior to entering clinical practice. Second, due to the retrospective nature of the study, we have no data on the microsatellite instability/mismatch repair status and somatic alterations (i.e., *KRAS*, *NRAS*, and *BRAF* mutations) of tumors, which are important markers to discriminate colorectal cancer patients with a different response to therapy and prognosis [14,15,16]. The lack of this information could have affected the interpretation of the results. Third, another limitation is the lack of phenotypic characterization of *IL15RA*-rs7910212 and *SMAD3*-rs7179840 polymorphisms by the functional assay that would have supported our findings. To fill this gap, a bioinformatic analysis was performed. However, if preliminary in silico data and literature evidences support a potential functional impact, the exact phenotypic consequences of *IL15RA*-rs7910212 and *SMAD3*-rs7179840 variants are still unknown, and further formal functional analyses are required to better understand the molecular mechanism underlying the observed associations.

## 5. Conclusions

In conclusion, the present study identified novel genetic prognostic markers, the *IL15RA*-rs7910212 and *SMAD3*-rs7179840 polymorphisms, which resulted in being significantly associated with OS in mCRC patients treated with the first-line FOLFIRI regimen. A prognostic score integrating four independent genetic variants (*IL15RA*-rs7910212, *SMAD3*-rs7179840, *NR1I2*-rs1054190, *VDR*-rs7299460) related to inflammation and immune response was also developed and demonstrated to stratify patients according to the different risks of death. These findings highlighted the relevance of genetic markers in the immune system and correlated pathways in predicting the effectiveness of chemotherapy and in identifying patients who are most likely to benefit from FOLFIRI administration. New insights into the role of inherited immune-related variants in modulating the interaction between chemotherapy and the immune system could also be of great interest considering the recent recognition of the therapeutic potential of chemotherapy and immunotherapy combination [65].

Systemic therapy for mCRC patients typically includes a chemotherapy backbone (i.e., 5-FU, irinotecan or oxaliplatin) paired with a targeted agent (antiangiogenics or anti-EGFR agents) into a two-drug or three-drug regimen. A three-drug regimen could represent a good choice to treat tumors at high risk of progression, but the cost in terms of the patients’ toxicity is sometimes very high. Hence, the selection of the more appropriate first-line therapeutic options becomes a complex issue influencing not only the course of therapy and patient survival but also safety and quality of life [14]. The selection of the type and sequence of treatments is currently based mainly on practice guidelines, patients’ status (e.g., age, performance status, comorbidities), disease features (e.g., respectability, tumor biology, tumor burden, clinical evolution) and therapy characteristics (e.g., toxicities, availability, costs). The contribution of additional selection criteria, as the host genetic profile, could improve the clinical decision-making and therapeutic planning. As an example, mCRC patients predicted to not benefit from FOLFIRI administration, based on their molecular profile, could be a candidate to receive an alternative regimen (i.e., FOLFOX) or an intensified treatment (FOLFOXIRI, 5-FU, irinotecan, oxaliplatin, leucovorin), thus increasing the chance of treatment efficacy. The capacity to select the most effective anti-cancer treatment has the potential to improve the management of patients with mCRC, not only with an increase in survival and quality of life but also with a concomitant reduction in medical costs.

## Figures and Tables

**Table 1:** Demographic and clinical characteristics of study populations (discovery cohort, *n* = 243 ^a^; replication cohort, *n* = 92).

	Discovery		Replication		
	N	(%)	N	(%)	
Gender					
Male	158	(65.0)	61	(66.3)	
Female	85	(35.0)	31	(33.7)	*p* = 0.8255
Age (years)					
<55	61	(25.1)	23	(25.0)	
55–59	32	(13.2)	18	(19.6)	
60–64	52	(21.4)	17	(18.5)	
≥65	98	(40.3)	34	(37.0)	*p* = 0.5128
Cancer site					
Right colon	76	(31.3)	23	(25.0)	
Left colon/Rectum	167	(68.7)	61	(66.3)	
Colon, NOS	0	(0.0)	8	(8.7)	*p* = 0.5030 ^b^
Stage at cancer diagnosis					
I–II	24	(9.9)			
III	64	(26.3)			
IV	155	(63.8)			
Radical surgery					
No	50	(20.6)			
Yes	193	(79.4)			
Adjuvant therapy					
No	163	(67.1)			
Yes	80	(32.9)			
Overall survival (95% CI)					
1 year	74.5% (68.5–80.0%)		72.1% (61.3–80.3%)		
2 years	41.6% (34.0–48.9%)		41.9% (31.4–52.0%)		
3 years	26.5% (18.4–35.4%)		20.1% (13.1–30.0%)		
5 years	9.2% (1.1–28.6%)		8.1% (3.6–15.1%)		*p* = 0.4867

^a^ Seven patients with polymorphisms frequency < 90% were excluded. ^b^ Excluding colon NOS. Abbreviation: NOS: Not Otherwise Specified.

**Table 2:** Genetic markers and overall survival in the discovery (*n* = 243) and replication (*n* = 92) cohorts. Only the associations with *p*-value < 0.05 and Bootstrap *p*-value < 0.05 are reported for the discovery cohort. Replicated markers are in bold.

				Discovery Cohort				Replication Cohort	
Genes	SNP	Base Change	Model	HR (95% CI) ^a^	*p*-Value	Bootstrap		HR (95% CI) ^b^	*p*-Value ^c^
						HR	*p*-Value		
*FAS*	rs983751	G > T	Dominant	1.60 (1.03–2.47)	0.0366	1.65	0.0319	1.82 (0.52–6.40)	0.1760
*FAS*	rs9658706	A > G	Dominant	1.84 (1.18–2.87)	0.0075	1.88	0.0094	0.88 (0.42–1.85)	
*FOXO3*	rs9384683	T > G	Recessive	4.39 (1.31–14.67)	0.0163	4.53	0.0185	---	---
*MIF*	rs738806	G > A	Dominant	1.55 (1.08–2.22)	0.0184	1.56	0.0217	1.06 (0.65–1.72)	0.4044
*IFNGR2*	rs1532	C > T	Dominant	0.53 (0.37–0.75)	0.0005	0.51	0.0005	1.05 (0.66–1.67)	
*IFNGR2*	rs9808753	A > G	Additive	1.57 (1.06–2.33)	0.0249	1.66	0.0165	1.13 (0.69–1.83)	0.3159
*IL15RA*	rs1998521	G > A	Additive	0.72 (0.54–0.95)	0.0211	0.71	0.0215	1.03 (0.73–1.46)	
*IL15RA*	rs2228059	A > C	Additive	1.45 (1.12–1.88)	0.0051	1.49	0.0042	0.99 (0.71–1.39)	
*IL15RA*	rs3136626	T > C	Dominant	1.56 (1.07–2.26)	0.0196	1.60	0.0169	1.02 (0.64–1.63)	0.4639
***IL15RA***	**rs7910212**	**T > C**	**Dominant**	**1.57 (1.04–2.39)**	**0.0327**	**1.62**	**0.0280**	**1.71 (0.93–3.12)**	**0.0411**
*SMAD3*	rs11636161	G > A	Additive	1.32 (1.03–1.70)	0.0282	1.34	0.0297	1.27 (0.89–1.80)	0.0963
*SMAD3*	rs1545161	T > C	Dominant	0.57 (0.40–0.83)	0.0029	0.56	0.0036	0.79 (0.48–1.31)	0.1809
*SMAD3*	rs3743343	T > C	Recessive	3.72 (1.10–12.55)	0.0345	3.79	0.0479	0.42 (0.08–2.23)	
***SMAD3***	**rs7179840**	**T > C**	**Dominant**	**0.65 (0.45–0.93)**	**0.0202**	**0.64**	**0.0203**	**0.61 (0.37–0.99)**	**0.0216**
*SMAD3*	rs718663	A > G	Dominant	1.75 (1.03–2.97)	0.0391	1.76	0.0502	1.43 (0.77–2.64)	0.1280
*STAT3*	rs17405722	G > A	Recessive	35.31 (4.14–300.87)	0.0011	39.62	0.0015	0.78 (0.10–6.09)	
*STAT3*	rs3744483	T > C	Dominant	0.61 (0.41–0.90)	0.0125	0.60	0.0144	1.23 (0.74–2.05)	
*STAT5A*	rs7217728	T > C	Dominant	0.69 (0.48–0.99)	0.0463	0.67	0.0404	0.95 (0.61–1.49)	0.4129
*STAT6*	rs167769	C > T	Dominant	1.81 (1.25–2.64)	0.0019	1.87	0.0019	0.97 (0.59–1.60)	
*TGFBR2*	rs12487185	A > G	Dominant	0.63 (0.44–0.92)	0.0152	0.64	0.0226	1.03 (0.64–1.65)	
*TGFBR2*	rs4583693	T > C	Recessive	3.14 (1.47–6.71)	0.0031	3.33	0.0027	1.36 (0.40–4.56)	0.3115
*TGFBR2*	rs5020833	C > G	Dominant	0.62 (0.43–0.90)	0.0125	0.62	0.0154	1.16 (0.72–1.85)	
*TLR10*	rs11466657	T > C	Additive	0.51 (0.30–0.88)	0.0206	0.50	0.0159	0.97 (0.28–3.41)	0.4822

Abbreviations: CI: Confidence interval; HR: Hazard ratios; SNP: Polymorphism. ^a^ Estimated from the Cox model, adjusted for age, sex, tumor stage at diagnosis, cancer site, radical surgery, and adjuvant chemotherapy. ^b^ Estimated from the Cox model, adjusted for age, sex, and cancer site. ^c^ Estimated from the one-tailed Wald χ^2^ test. Some clinical data available for the discovery cohort (i.e., radical surgery, adjuvant chemotherapy, tumor stage at diagnosis), were not available for the replication cohort.

**Table 3:** Construction of a prognostic score for overall survival using selected polymorphisms (SNPs) in the combined case series of 320 patients with available SNPs.

Gene-SNP	HR (95% CI) ^a^	HR (95% CI) ^b^	Score Points
*IL15RA*-rs7910212 (TC/CC vs. TT)	1.55 (1.12–2.15)	1.66 (1.19–2.31)	1
*SMAD3*-rs7179840 (TT vs. TC/CC)	1.53 (1.15–2.04)	1.54 (1.15–2.06)	1
*VDR*-rs7299460 (CC vs. CT/TT)	1.53 (1.15–2.03)	1.48 (1.10–1.99)	1
*NR1I2*-rs1054190 (TT vs. CC/CT)	4.61 (1.97–10.81)	4.31 (1.82–10.20)	4

^a^ Estimated from the Cox proportional hazard model, conditioned on cohort, and adjusted for gender, age, cancer site, and radical surgery (when available). ^b^ Further adjusted for potential SNP-SNP interaction.

**Table 4:** Hazard ratios (HR) and 95% confidence interval (CI) for overall survival in the 320 patients with mCRC according to the genetic score.

Genetic Score				Genetic Score (Combined Categories)				
Score Points	Patients		HR (95% CI) ^a^	Score Points	Patients		HR (95% CI) ^a^	*p*-Value
	*n*	(%)			*n*	(%)		
0	77	(24.1)	Reference	0	77	(24.1)	Reference	
1	129	(40.3)	1.72 (1.15–2.57)	1–2	223	(69.7)	1.90 (1.31–2.76)	0.0007
2	94	(29.4)	2.21 (1.45–3.38)					
3	12	(3.8)	6.93 (3.34–14.39)	≥3	20	(6.3)	7.37 (3.93–13.84)	<0.0001
≥4	8	(2.5)	8.57 (3.43–21.36)					

^a^ Estimated from the Cox proportional hazard model, conditioned on cohort, and adjusted for gender, age, cancer site, and radical surgery (when available).

## Data Availability

The data presented in this study are available on request from the corresponding author. The data are not publicly available due to ethical restrictions.

## Supplementary Materials

The following are available online at https://www.mdpi.com/article/10.3390/cancers13071705/s1. Table S1: Candidate genes and related tagging polymorphisms selected for the pharmacogenetic analysis; Table S2: Distribution of metastatic colorectal cancer patients from the discovery (*n* = 243) and replication (*n* = 92) cohorts according to relevant gene polymorphisms (SNP); Table S3: In silico predicted functional effect of polymorphisms in the A) *IL15RA*-rs7910212 and B) *SMAD3-*rs7179840 haploblocks by HaploReg v.4.1, RegulomeDB v2.0, and Ensembl’s Variant Effect Predictor (VEP) Ensembl GRCh37 release 102–November 2020. Only the most relevant data are reported in the Table. The targeted marker is in bold.

## References

[1] Di Caro G., Marchesi F., Laghi L., Grizzi F. (2013). Immune cells: Plastic players along colorectal cancer progression. J. Cell. Mol. Med..

[2] Wang Y.-J., Fletcher R., Yu J., Zhang L. (2018). Immunogenic effects of chemotherapy-induced tumor cell death. Genes Dis..

[3] Asleh K., Brauer H.A., Sullivan A., Lauttia S., Lindman H., Nielsen T.O., Joensuu H., Thompson E.A., Chumsri S. (2020). Predictive Biomarkers for Adjuvant Capecitabine Benefit in Early-Stage Triple-Negative Breast Cancer in the FinXX Clinical Trial. Clin. Cancer Res..

[4] Gmeiner W.H. (2020). Fluoropyrimidine Modulation of the Anti-Tumor Immune Response―Prospects for Improved Colorectal Cancer Treatment. Cancers.

[5] Kanterman J., Sade-Feldman M., Biton M., Ish-Shalom E., Lasry A., Goldshtein A., Hubert A., Baniyash M. (2014). Adverse Immunoregulatory Effects of 5FU and CPT11 Chemotherapy on Myeloid-Derived Suppressor Cells and Colorectal Cancer Outcomes. Cancer Res..

[6] Maeda K., Hazama S., Tokuno K., Kan S., Maeda Y., Watanabe Y., Kamei R., Shindo Y., Maeda N., Yoshimura K. (2011). Impact of chemotherapy for colorectal cancer on regulatory T-cells and tumor immunity. Anticancer Res..

[7] Vincent J., Mignot G., Chalmin F., Ladoire S., Bruchard M., Chevriaux A., Martin F., Apetoh L., Rébé C., Ghiringhelli F. (2010). 5-Fluorouracil Selectively Kills Tumor-Associated Myeloid-Derived Suppressor Cells Resulting in Enhanced T Cell–Dependent Antitumor Immunity. Cancer Res..

[8] Cecchin E., De Mattia E., Toffoli G. (2016). Nuclear receptors and drug metabolism for the personalization of cancer therapy. Expert Opin. Drug Metab. Toxicol..

[9] De Mattia E., Cecchin E., Roncato R., Toffoli G. (2016). Pregnane X receptor, constitutive androstane receptor and hepatocyte nuclear factors as emerging players in cancer precision medicine. Pharmacogenomics.

[10] De Mattia E., Dreussi E., Cecchin E., Toffoli G. (2013). Pharmacogenetics of the nuclear hormone receptors: The missing link between environment and drug effects?. Pharmacogenomics.

[11] De Mattia E., Cecchin E., Montico M., Labriet A., Guillemette C., Dreussi E., Roncato R., Bignucolo A., Buonadonna A., D’Andrea M. (2018). Association of STAT-3 rs1053004 and VDR rs11574077 with FOLFIRI-Related Gastrointestinal Toxicity in Metastatic Colorectal Cancer Patients. Front. Pharmacol..

[12] De Mattia E., Polesel J., Roncato R., Labriet A., Bignucolo A., Dreussi E., Romanato L., Guardascione M., Buonadonna A., D’Andrea M. (2019). Germline Polymorphisms in the Nuclear Receptors PXR and VDR as Novel Prognostic Markers in Metastatic Colorectal Cancer Patients Treated With FOLFIRI. Front. Oncol..

[13] Labriet A., De Mattia E., Cecchin E., Lévesque É., Jonker D., Couture F., Buonadonna A., D’Andrea M., Villeneuve L., Toffoli G. (2017). Improved Progression-Free Survival in Irinotecan-Treated Metastatic Colorectal Cancer Patients Carrying the HNF1A Coding Variant p.I27L. Front. Pharmacol..

[14] Dekker E., Tanis P.J., Vleugels J.L.A., Kasi P.M., Wallace M.B. (2019). Colorectal cancer. Lancet.

[15] Gherman A., Balacescu L., Gheorghe-Cetean S., Vlad C., Balacescu O., Irimie A., Lisencu C. (2020). Current and New Predictors for Treatment Response in Metastatic Colorectal Cancer. The Role of Circulating miRNAs as Biomarkers. Int. J. Mol. Sci..

[16] Xie Y.-H., Chen Y.-X., Fang J.-Y. (2020). Comprehensive review of targeted therapy for colorectal cancer. Signal Transduct. Target. Ther..

[17] Gil-Delgado M.A., Guinet F., Castaing D., Adam R., Coeffic D., Durrani A.K.S., Bismuth H., Khayat D. (2001). Prospective Phase II Trial of Irinotecan, 5-Fluorouracil, and Leucovorin in Combination as Salvage Therapy for Advanced Colorectal Cancer. Am. J. Clin. Oncol..

[18] Saltz L.B., Douillard J., Pirotta N., Alakl M., Gruia G., Awad L., Elfring G.L., Locker P.K., Miller L.L. (2001). Irinotecan Plus Fluorouracil/Leucovorin for Metastatic Colorectal Cancer: A New Survival Standard. Oncology.

[19] Cecchin E., De Mattia E., Ecca F., Toffoli G. (2018). Host genetic profiling to increase drug safety in colorectal cancer from discovery to implementation. Drug Resist. Updat..

[20] De Mattia E., Cecchin E., Toffoli G. (2015). Pharmacogenomics of intrinsic and acquired pharmacoresistance in colorectal cancer: Toward targeted personalized therapy. Drug Resist. Updat..

[21] Olivera G., Sendra L., Herrero M.J., Puig C., Aliño S.F. (2019). Colorectal cancer: Pharmacogenetics support for the correct drug prescription. Pharmacogenomics.

[22] Moradi-Marjaneh R., Khazaei M., Seifi S., Hassanian S.M., Ferns G.A., Avan A. (2018). Pharmacogenetics of Anticancer Drug Sensitivity and Toxicity in Colorectal Cancer. Curr. Pharm. Des..

[23] Palmirotta R., Carella C., Silvestris E., Cives M., Stucci S.L., Tucci M., Lovero D., Silvestris F. (2018). SNPs in predicting clinical efficacy and toxicity of chemotherapy: Walking through the quicksand. Oncotarget.

[24] Jia J., Zhang P., Gou M., Yang F., Qian N., Dai G. (2019). The Role of Serum CEA and CA19-9 in Efficacy Evaluations and Progression-Free Survival Predictions for Patients Treated with Cetuximab Combined with FOLFOX4 or FOLFIRI as a First-Line Treatment for Advanced Colorectal Cancer. Dis. Markers.

[25] Suenaga M., Matsusaka S., Ueno M., Yamamoto N., Shinozaki E., Mizunuma N., Yamaguchi T., Hatake K. (2011). Predictors of the efficacy of FOLFIRI plus bevacizumab as second-line treatment in metastatic colorectal cancer patients. Surg. Today.

[26] Shen H., Yang J., Huang Q., Jiang M.-J., Tan Y.-N., Fu J.-F., Zhu L.-Z., Fang X.-F., Yuan Y. (2015). Different treatment strategies and molecular features between right-sided and left-sided colon cancers. World J. Gastroenterol..

[27] Cecchin E., Innocenti F., D’Andrea M., Corona G., De Mattia E., Biason P., Buonadonna A., Toffoli G. (2009). Predictive Role of the UGT1A1, UGT1A7, and UGT1A9 Genetic Variants and Their Haplotypes on the Outcome of Metastatic Colorectal Cancer Patients Treated with Fluorouracil, Leucovorin, and Irinotecan. J. Clin. Oncol..

[28] Toffoli G., Cecchin E., Corona G., Russo A., Buonadonna A., D’Andrea M., Pasetto L.M., Pessa S., Errante D., De Pangher V. (2006). The Role of UGT1A1*28 Polymorphism in the Pharmacodynamics and Pharmacokinetics of Irinotecan in Patients with Metastatic Colorectal Cancer. J. Clin. Oncol..

[29] Tournigand C., André T., Achille E., Lledo G., Flesh M., Mery-Mignard D., Quinaux E., Couteau C., Buyse M., Ganem G. (2004). FOLFIRI Followed by FOLFOX6 or the Reverse Sequence in Advanced Colorectal Cancer: A Randomized GERCOR Study. J. Clin. Oncol..

[30] Lévesque É., Bélanger A.-S., Harvey M., Couture F., Jonker D., Innocenti F., Cecchin E., Toffoli G., Guillemette C. (2013). Refining theUGT1AHaplotype Associated with Irinotecan-Induced Hematological Toxicity in Metastatic Colorectal Cancer Patients Treated with 5-Fluorouracil/Irinotecan-Based Regimens. J. Pharmacol. Exp. Ther..

[31] De Mattia E., Dreussi E., Montico M., Gagno S., Zanusso C., Quartuccio L., De Vita S., Guardascione M., Buonadonna A., D’Andrea M. (2018). A Clinical-Genetic Score to Identify Surgically Resected Colorectal Cancer Patients Benefiting From an Adjuvant Fluoropyrimidine-Based Therapy. Front. Pharmacol..

[32] Illumina Website. https://illumina.com.

[33] HaploReg v4.1. https://pubs.broadinstitute.org/mammals/haploreg/haploreg.php.

[34] RegulomeDB v2.0. https://regulomedb.org/regulome-search/).

[35] Ensembl’s Variant Effect Predictor. https://www.ensembl.org/info/docs/tools/vep/index.html).

[36] Rentzsch P., Witten D., Cooper G.M., Shendure J., Kircher M. (2019). CADD: Predicting the deleteriousness of variants throughout the human genome. Nucleic Acids Res..

[37] National Center for Biotechnology Information (NCBI)-dbSNP Database. http://www.ncbi.nlm.nih.gov/snp.

[38] Fumagalli M., Pozzoli U., Cagliani R., Comi G.P., Riva S., Clerici M., Bresolin N., Sironi M. (2009). Parasites represent a major selective force for interleukin genes and shape the genetic predisposition to autoimmune conditions. J. Exp. Med..

[39] Grizzi F., Bianchi P., Malesci A., Laghi L. (2013). Prognostic value of innate and adaptive immunity in colorectal cancer. World J. Gastroenterol..

[40] Lasry A., Zinger A., Ben-Neriah A.L.A.Z.Y. (2016). Inflammatory networks underlying colorectal cancer. Nat. Immunol..

[41] Markman J.L., Shiao S.L. (2015). Impact of the immune system and immunotherapy in colorectal cancer. J. Gastrointest. Oncol..

[42] Tuomisto E.A., Mäkinen M.J., Väyrynen J.P. (2019). Systemic inflammation in colorectal cancer: Underlying factors, effects, and prognostic significance. World J. Gastroenterol..

[43] Steel J.C., Waldmann T.A., Morris J.C. (2012). Interleukin-15 biology and its therapeutic implications in cancer. Trends Pharmacol. Sci..

[44] Guo Y., Luan L., Patil N.K., Sherwood E.R. (2017). Immunobiology of the IL-15/IL-15Rα complex as an antitumor and antiviral agent. Cytokine Growth Factor Rev..

[45] Bergh J.M.V.D., Lion E., Van Tendeloo V.F., Smits E.L. (2017). IL-15 receptor alpha as the magic wand to boost the success of IL-15 antitumor therapies: The upswing of IL-15 transpresentation. Pharmacol. Ther..

[46] Cui F., Qu D., Sun R., Zhang M., Nan K. (2019). NK cell-produced IFN-γ regulates cell growth and apoptosis of colorectal cancer by regulating IL-15. Exp. Ther. Med..

[47] Yu P., Steel J.C., Zhang M., Morris J.C., Waldmann T.A. (2010). Simultaneous Blockade of Multiple Immune System Inhibitory Checkpoints Enhances Antitumor Activity Mediated by Interleukin-15 in a Murine Metastatic Colon Carcinoma Model. Clin. Cancer Res..

[48] Kobayashi H., Dubois S., Sato N., Sabzevari H., Sakai Y., Waldmann T.A., Tagaya Y. (2005). Role of trans-cellular IL-15 presentation in the activation of NK cell–mediated killing, which leads to enhanced tumor immunosurveillance. Blood.

[49] Cao S., Black J.D., Troutt A.B., Rustum Y.M. (1998). Interleukin 15 offers selective protection from irinotecan-induced intestinal toxicity in a preclinical animal model. Cancer Res..

[50] Cao S., Troutt A.B., Rustum Y.M. (1998). Interleukin 15 protects against toxicity and potentiates antitumor activity of 5-fluorouracil alone and in combination with leucovorin in rats bearing colorectal cancer. Cancer Res..

[51] Shinohara H., Bucana C.D., Killion J.J., Fidler I.J. (2000). Intensified Regression of Colon Cancer Liver Metastases in Mice Treated with Irinotecan and the Immunomodulator JBT 3002. J. Immunother..

[52] Koveitypour Z., Panahi F., Vakilian M., Peymani M., Forootan F.S., Esfahani M.H.N., Ghaedi K. (2019). Signaling pathways involved in colorectal cancer progression. Cell Biosci..

[53] Tauriello D.V.F., Batlle E. (2016). Targeting the Microenvironment in Advanced Colorectal Cancer. Trends Cancer.

[54] Liu X., Ji Q., Fan Z., Li Q. (2015). Cellular signaling pathways implicated in metastasis of colorectal cancer and the associated targeted agents. Future Oncol..

[55] Romano G., Santi L., Bianco M.R., Giuffrè M.R., Pettinato M., Bugarin C., Garanzini C., Savarese L., Leoni S., Cerrito M.G. (2016). The TGF-β pathway is activated by 5-fluorouracil treatment in drug resistant colorectal carcinoma cells. Oncotarget.

[56] Jung B., Staudacher J.J., Beauchamp D. (2017). Transforming Growth Factor β Superfamily Signaling in Development of Colorectal Cancer. Gastroenterology.

[57] Slattery M.L., Herrick J.S., Lundgreen A., Wolff R.K. (2011). Genetic Variation in the TGF-β Signaling Pathway and Colon and Rectal Cancer Risk. Cancer Epidemiol. Biomarkers Prev..

[58] Slattery M.L., Lundgreen A. (2014). The Influence of the CHIEF Pathway on Colorectal Cancer-Specific Mortality. PLoS ONE.

[59] Huang M.-Y., Lin C.-H., Huang C.-M., Tsai H.-L., Huang C.-W., Yeh Y.-S., Chai C.-Y., Wang J.-Y. (2015). Relationships Between SMAD3 Expression and Preoperative Fluoropyrimidine-Based Chemoradiotherapy Response in Locally Advanced Rectal Cancer Patients. World J. Surg..

[60] Moon S.U., Kang M.H., Sung J.H., Kim J.W., Lee J.O., Kim Y.J., Lee K.W., Bang S.M., Lee J.S., Kim J.H. (2014). Effect of Smad3/4 on chemotherapeutic drug sensitivity in colorectal cancer cells. Oncol. Rep..

[61] Juliá E.P., Mordoh J., Levy E.M. (2020). Cetuximab and IL-15 Promote NK and Dendritic Cell Activation In Vitro in Triple Negative Breast Cancer. Cells.

[62] Pinette A., McMichael E., Courtney N.B., Duggan M., Benner B.N., Choueiry F., Yu L., Abood D., Mace T.A., Carson W.E. (2019). An IL-15-based superagonist ALT-803 enhances the NK cell response to cetuximab-treated squamous cell carcinoma of the head and neck. Cancer Immunol. Immunother..

[63] Rocca Y.S., Roberti M.P., Juliá E.P., Pampena M.B., Bruno L., Rivero S., Huertas E., Loria F.S., Pairola A., Caignard A. (2016). Phenotypic and Functional Dysregulated Blood NK Cells in Colorectal Cancer Patients Can Be Activated by Cetuximab Plus IL-2 or IL-15. Front. Immunol..

[64] Yegodayev K.M., Novoplansky O., Golden A., Prasad M., Levin L., Jagadeeshan S., Zorea J., Dimitstein O., Joshua B.-Z., Cohen L. (2020). TGF-Beta-Activated Cancer-Associated Fibroblasts Limit Cetuximab Efficacy in Preclinical Models of Head and Neck Cancer. Cancers.

[65] Yu W.-D., Sun G., Li J., Xu J., Wang X. (2019). Mechanisms and therapeutic potentials of cancer immunotherapy in combination with radiotherapy and/or chemotherapy. Cancer Lett..

