# Supplementary material for: IL15RA and SMAD3 Genetic Variants Predict Overall Survival in Metastatic Colorectal Cancer Patients Treated with FOLFIRI Therapy: A New Paradigm
Source: Cancers (Basel). 2021 Apr 3;13(7):1705. doi: 10.3390/cancers13071705 (PMC8038482; doi:10.3390/cancers13071705)
Supplement: Supplementary file 1 [file cancers-13-01705-s001.pdf]

Supplementary Table S1: Candidate genes and related Tagging polymorphisms selected for pharmacogenetic analysis.

Gene	Tagging polymorphisms
CD276 (B7-H3)	rs3825859
	rs8038465
	rs2127015
	rs10083681
CXCR7	rs10179774
	rs7559855
	rs34135799
	rs10184764
FAS	rs3740286
	rs7097467
	rs1800682
	rs2234978
	rs9658727
	rs4406737
	rs9658706
	rs982764
	rs983751
FOXO3	rs2153960
	rs12203787
	rs7762395
	rs2802288
	rs9384683
	rs9486902
	rs13207511
	rs12196996
	rs1536057
	rs3800230
	rs7746906
	rs2294019
	rs2232365
	rs3761548
	rs3761547
IFNG	rs1861494
IFNGR1	rs9376269
	rs10457655
IFNGR2	rs9808685
	rs9808753
	rs1532
	rs2834213
	rs2834211
IL15RA	rs17320853
	rs8177613

	rs8177633
	rs8177654
	rs2296141
	rs1998521
	rs3136626
	rs2228059
	rs7910212
	rs3736862
IL17A	rs1892280
	rs2275913
	rs10484879
IL17F	rs607175
	rs12210153
	rs641701
	rs2064331
	rs9463772
	rs763780
IL2RA	rs12722489
	rs1107345
	rs706778
	rs10905656
	rs2256774
	rs6602398
	rs12722588
	rs3118470
	rs11256448
	rs10905668
	rs4749920
IL2RB	rs2284033
	rs3218266
	rs84460
	rs3218322
	rs3218258
	rs228942
IL2RG	rs12857595
IL8	rs2227306
MIF	rs2000466
	rs738806
	rs875643
	rs1007888
MMP3	rs679620
	rs569444
	rs683878
PRDM1	rs4946722
	rs1984224

	rs573869
	rs811925
	rs6923608
SMAD3	rs2118613
	rs11636161
	rs17293632
	rs12917612
	rs4776338
	rs2033785
	rs17228212
	rs4776343
	rs12708492
	rs1545161
	rs4147358
	rs3743343
	rs12916733
	rs718663
	rs2033787
	rs2289263
	rs12914140
	rs16950635
	rs991157
	rs7162912
	rs7179840
	rs4776887
	rs9302242
SMAD3	rs11856909
SMAD4	rs12457540
	rs948588
	rs10502913
STAT3	rs3744483
	rs9891119
	rs8069645
	rs744166
	rs17405722
STAT5A	rs1053023
	rs7217728
STAT5B	rs8080122
STAT6	rs703817
	rs3024979
	rs3024974
	rs1059513
	rs167769
TGFBR1	rs10988716
	rs928180

TGFBR2	rs3773632
	rs1841528
	rs5020833
	rs2276767
	rs3773658
	rs9867701
	rs12487185
	rs9790268
	rs4955104
	rs4583693
	rs995435
	rs3773649
	rs6550004
	rs4955212
	rs764522
	rs1346907
	rs876688
	rs4522809
	rs1078985
	rs3773662
	rs17025857
	rs11709624
	rs11924422
	rs1835538
	rs9310940
	rs1991657
TIMP1	rs6609533
	rs6609534
TIRAP	rs8177376
	rs10893493
	rs625413
	rs1893352
	rs1786704
TLR10	rs11466617
	rs7660429
	rs11096955
	rs11096957
	rs11725309
	rs11466657
TLR3	rs11721827
	rs5743303
	rs7657186
	rs3775291
TLR4	rs1927911
	rs11536898

	rs1927906
	rs7037117
	rs5030717
	rs12377632
	rs4986791
TLR6	rs7673124
	rs1039559
	rs2174284
VEGFA	rs833069
	rs3025033
	rs699947
	rs2146323
WNT5A	rs1829556
	rs11706227
	rs524153

Supplementary Table S2: Distribution of patients with metastatic colorectal cancer from the discovery (n=243) and replication (n=92) cohorts according to relevant gene polymorphisms (SNP).

Genes	SNP	Base change	Discovery cohort				Replication cohort			
			Genotype frequency			HW equilibrium[a]	Genotype frequency			HW equilibrium[a]
			AA	Aa	aa		AA	Aa	aa	
FAS	rs983751	G>T	0.810	0.188	0.012	p=0.7135	0.744	0.211	0.044	p=0.1025
FAS	rs9658706	A>G	0.815	0.181	0.004	p=0.3783	0.848	0.152	0.000	p=0.4239
FOXO3	rs9384683	T>G	0.826	0.162	0.012	p=0.4937	0.859	0.141	0.000	p=0.4659
MIF	rs738806	G>A	0.527	0.416	0.058	p=0.3046	0.571	0.352	0.077	p=0.5101
IFNGR2	rs1532	C>T	0.465	0.428	0.107	p=0.7769	0.500	0.413	0.087	p=0.9695
IFNGR2	rs9808753	A>G	0.778	0.214	0.008	p=0.4403	0.739	0.250	0.011	p=0.5352
IL15RA	rs1998521	G>A	0.259	0.556	0.185	p=0.0676	0.304	0.457	0.239	p=0.4257
IL15RA	rs2228059	A>C	0.259	0.535	0.206	p=0.2594	0.272	0.478	0.250	p=0.6798
IL15RA	rs3136626	T>C	0.473	0.449	0.078	p=0.3258	0.565	0.304	0.130	p=0.0167
IL15RA	rs7910212	T>C	0.770	0.214	0.017	p=0.8606	0.816	0.172	0.012	p=0.8366
SMAD3	rs11636161	G>A	0.432	0.420	0.148	p=0.1757	0.467	0.435	0.098	p=0.9456
SMAD3	rs1545161	T>C	0.357	0.469	0.174	p=0.6425	0.337	0.511	0.152	p=0.5789
SMAD3	rs3743343	T>C	0.616	0.372	0.012	p=0.0084	0.609	0.348	0.044	p=0.8312
SMAD3	rs7179840	T>C	0.500	0.391	0.109	p=0.2497	0.337	0.533	0.130	p=0.2798
SMAD3	rs718663	A>G	0.872	0.128	0.000	p=0.2882	0.837	0.152	0.011	p=0.6894
STAT3	rs17405722	G>A	0.893	0.103	0.004	p=0.7599	0.815	0.174	0.011	p=0.8877
STAT3	rs3744483	T>C	0.634	0.321	0.045	p=0.7799	0.663	0.272	0.065	p=0.1390
STAT5A	rs7217728	T>C	0.449	0.440	0.111	p=0.9233	0.446	0.457	0.098	p=0.7104
STAT6	rs167769	C>T	0.496	0.401	0.103	p=0.4159	0.380	0.533	0.087	p=0.1122
TGFBR2	rs12487185	A>G	0.531	0.403	0.066	p=0.6498	0.389	0.511	0.100	p=0.2740
TGFBR2	rs4583693	T>C	0.626	0.329	0.045	p=0.9087	0.533	0.485	0.033	p=0.1262
TGFBR2	rs5020833	C>G	0.527	0.398	0.075	p=0.9872	0.402	0.500	0.098	p=0.3275
TLR10	rs11466657	T>C	0.844	0.148	0.008	p=0.7637	0.967	0.033	0.000	p=0.8737

Abbreviations: HW, Hardy–Weinberg.

[a] Deviation from Hardy–Weinberg equilibrium was tested by chi-squared test, and deviation was considered at $P<0.05$.

Supplementary Table S3: *In silico* predicted functional effect of polymorphisms in the **A)** *IL15RA*-rs7910212 and **B)** *SMAD3*-rs7179840 haploblocks by HaploReg v.4.1 , RegulomeDB v2.0 and Ensembl's Variant Effect Predictor (VEP) Ensembl release 102 - November 2020. Only the most relevant data are reported in the Table. Targeted marker is bold.

A) *IL15RA-rs7910212*

General data from Haploreg and Ensembl's VEP						HaploReg&					Ensembl's VEP			RegulomeDB	
dbSNP ID (Haploblock by Haploreg)	Chromosome Location (GRCh38)	LD (r^2)	SNP Location	Consequence	Impact*	Promoter histone marks	Enhancer histone marks	DNAse	Motifs changed	GRASP QTL hits	PHRED-like scaled CADD score**	Associated Phenotypes	PubMed (PMID)	Rank^	Score^^
rs8177685	chr10: 10:5966650	0.97	IL15RA (intronic)	intron variant	Modifier		1 Tissue (FAT)		Pax-4,VDR		1.522		19468064, 20018074	5	0.45052
rs7917197	chr10: 5967063	0.96	IL15RA (intronic)	intron variant	Modifier				Homez		5.163			5	0.1708
**rs7910212**	**chr10: 10:5967163**		**IL15RA (intronic)**	**intron variant**	**Modifier**				**Hoxa7**		**2.490**			**5**	**0.13454**

B) *SMAD- rs7179840*

General data from Haploreg and Ensembl's VEP						HaploReg&					Ensembl's VEP			RegulomeDB	
dbSNP ID (Haploblock by Haploreg)	Chromosome Location (GRCh38)	LD (r^2)	SNP Location	Consequence	Impact*	Promoter histone marks	Enhancer histone marks	DNAse	Motifs changed	GRASP QTL hits	PHRED-like scaled CADD score**	Associated Phenotypes	PubMed (PMID)	Rank^	Score^^
**rs7179840**	**chr15:67166592**		**SMAD3 (intronic)**	**intron variant**	**Modifier**	**2 tissues (ESC, IPSC)**	**15 tissues (ESC, ESDR, LNG, IPSC, FAT, BRST, MUS, BRN, SKIN, VAS, GI, ADRL, HRT, OVRY, SPLN)**	**9 tissues (ESC,LNG,BLD,SKIN,HRT,OVRY,MUS,LNG,SKIN)**	**ERalpha-a,Hmx,Nkx2**		**0.126**	**yes**		**4**	**0.60906**
rs7183244	Chr15:67168973	0.97	SMAD3 (intronic)	intron variant	Modifier	1 tissue (MUS)	14 tissues (ESC, LNG, FAT, MUS, BRN, SKIN, ADRL, HRT, GI, KID, OVRY, PANC, VAS, BONE)	2 tissues (LNG,VAS)		1 hit	1.396	yes	21068203, 21984931	4	0.60906

& No data for: SiPhy cons, Proteins bound, NHGRI/EBI GWAS hits; Selected eQTL hits.

* Subjective impact classification of consequence type

** Score directly proportional to the variant deleteriousness (https://cadd.gs.washington.edu/info)

^ Rank score ranges from 1 to 7 with the lower value indicating the stronger evidence for a variant to be in a functional region. 4= TF binding + DNase peak; 5=TF binding or DNase peak.

^^ Probability score ranges from 0 to 1, with 1 being most likely to be a regulatory variant.

Abbreviation: LD, linkage disequilibrium; SNP, single nucleotide polymorphism; CADD, Combined Annotation Dependent Depletion.
